# Evolutionary flexibility and rigidity in the bacterial methylerythritol phosphate (MEP) pathway

**DOI:** 10.3389/fmicb.2023.1286626

**Published:** 2023-11-08

**Authors:** Bailey Marshall, Kaustubh Amritkar, Michael Wolfe, Betül Kaçar, Robert Landick

**Affiliations:** ^1^Department of Biochemistry, University of Wisconsin–Madison, Madison, WI, United States; ^2^DOE Great Lakes Bioenergy Research Center, University of Wisconsin–Madison, Madison, WI, United States; ^3^Department of Bacteriology, University of Wisconsin–Madison, Madison, WI, United States

**Keywords:** isoprenoid biosynthesis, terpenoid biosynthesis, metabolic engineering, synthetic biology, comparative genomics

## Abstract

Terpenoids are a diverse class of compounds with wide-ranging uses including as industrial solvents, pharmaceuticals, and fragrances. Efforts to produce terpenoids sustainably by engineering microbes for fermentation are ongoing, but industrial production still largely relies on nonrenewable sources. The methylerythritol phosphate (MEP) pathway generates terpenoid precursor molecules and includes the enzyme Dxs and two iron–sulfur cluster enzymes: IspG and IspH. IspG and IspH are rate limiting-enzymes of the MEP pathway but are challenging for metabolic engineering because they require iron–sulfur cluster biogenesis and an ongoing supply of reducing equivalents to function. Therefore, identifying novel alternatives to IspG and IspH has been an on-going effort to aid in metabolic engineering of terpenoid biosynthesis. We report here an analysis of the evolutionary diversity of terpenoid biosynthesis strategies as a resource for exploration of alternative terpenoid biosynthesis pathways. Using comparative genomics, we surveyed a database of 4,400 diverse bacterial species and found that some may have evolved alternatives to the first enzyme in the pathway, Dxs making it evolutionarily flexible. In contrast, we found that IspG and IspH are evolutionarily rigid because we could not identify any species that appear to have enzymatic routes that circumvent these enzymes. The ever-growing repository of sequenced bacterial genomes has great potential to provide metabolic engineers with alternative metabolic pathway solutions. With the current state of knowledge, we found that enzymes IspG and IspH are evolutionarily indispensable which informs both metabolic engineering efforts and our understanding of the evolution of terpenoid biosynthesis pathways.

## 1. Introduction

Terpenoids are a diverse class of biomolecules with varied cellular functions, ranging from basic roles such as maintaining membrane fluidity and supporting electron transport to highly specialized roles such as hormone signaling and cellular defense (Sell, 2006; Avalos et al., 2022). All terpenoids are built from five carbon-building block precursors [dimethylallyl diphosphate (DMADP) and isopentenyl diphosphate (IDP)] and exhibit high structural diversity with over 95,000 different terpenoid compounds identified to date (Faylo, 2021). With applications as diverse as their molecular structures, terpenoids are used extensively in support of modern-day life such as commodity solvents, pharmaceuticals, and fuels (Ajikumar et al., 2008; Tracy et al., 2009; Harvey et al., 2010; George et al., 2015) and for exploring life’s earliest record on Earth (Brocks et al., 1999; Summons et al., 2006; Hillier and Lathe, 2019).

Terpenoids are currently harvested from plants, generated by microbial fermentation, or produced chemically from nonrenewable petroleum (Leavell et al., 2016). To date, variable yields, inefficiencies, and cost have made natural terpenoid production from plants or microbes unattractive leading to a global terpenoid supply chain that favors production from petroleum at the expense of sustainability and environmental impact (Leavell et al., 2016). Engineered microbes have the potential for improved yields and increased diversity in the types of terpenoids that are produced, with some important successes to date (George et al., 2015; Benjamin et al., 2016; Navale et al., 2021). Current efforts in engineering microbes for terpenoid production use one or both of two characterized “canonical” biosynthetic pathways (the mevalonate [MEV] and methylerythritol phosphate [MEP] pathways) and aim to produce higher yields and an ever-increasing range of terpenoid products as sustainably and economically as possible. However, knowledge about the evolutionary flexibility of terpenoid biosynthesis or possible alternative metabolic routes for terpenoid biosynthesis is lacking.

Terpenoids are used in membranes in all known domains of life (Lange et al., 2000; Holstein and Hohl, 2004) and are present in molecular fossils billions of years old (Ourisson and Albrecht, 1992; Brocks et al., 1999, 2005). Given their universality and presence in the fossil records, terpenoids are identified as biomarkers, providing insight into life’s earliest evolution on Earth (Lange et al., 2000; Lombard and Moreira, 2011; Summons et al., 2022). Yet, important questions remain about the evolution of terpenoid biosynthesis, including the identity of the primordial pathway for terpenoid biosynthesis and how horizontal gene transfer may have contributed to the evolution of terpenoid biosynthetic pathways (Zeng and Dehesh, 2021; Hoshino and Villanueva, 2023). Greater understanding of the variations within terpenoid biosynthetic pathways may further elucidate the evolution of this important ancient pathway.

**Figure 1:**
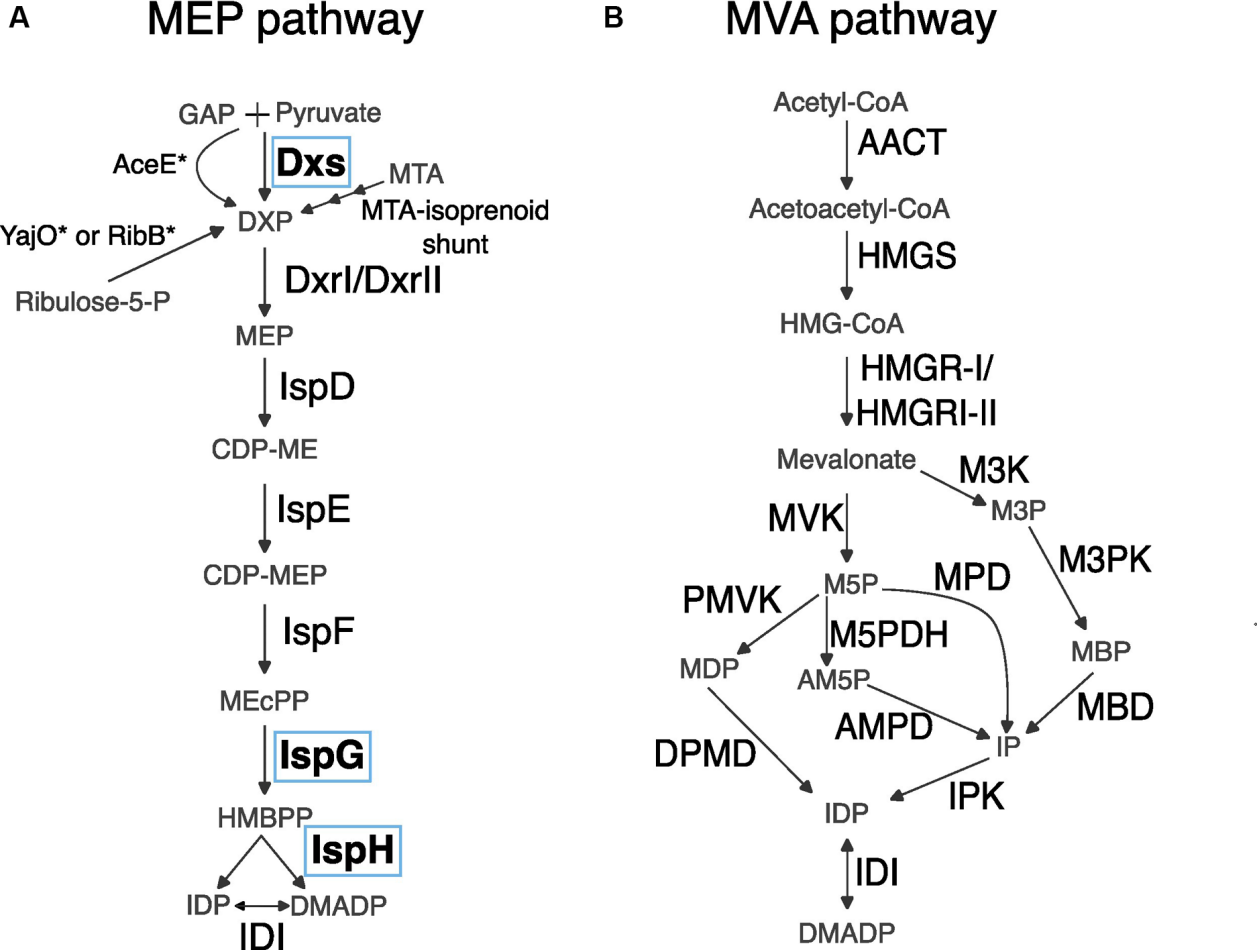
Reactions of MEP and MVA metabolic pathways. **(A)** MEP pathway. Asterisks indicate a mutant version of an enzyme is necessary for completing a reaction. Enzymes circled in blue, Dxs, IspG, and IspH, are the focus of this report. GAP, glyceraldehyde 3-phosphate; Dxs, DXP synthase; DXP, 1-deoxyxylulose 5-phosphate; Dxr, DXS reductoisomerase; MEP, 2-C-methyl-erythritol 4-phosphate; CDP-ME, 4-(cytidine 5′-diphospho)-2-C-methyl-erythritol; CDP-MEP, 2-phospho-4-(cytidine 5′-diphospho)-2-C-methyl-erythritol; MEcPP, 2-C-methyl-erythritol-2,4-cyclodiphosphate; HMBPP, 4-hydroxy-3-methyl 2-butenyl diphosphate; IDP, isopentenyl diphosphate; DMADP, dimethylallyl diphosphate. **(B)** MVA pathway. AACT, Acetoacetyl-CoA thiolase; HMGS, HMG synthase; HMG-CoA, hydroxymethylglutaryl-CoA; HMGR, HMG reductase; MVK, mevalonate kinase; M5P, mevalonate 5-phosphate; M3K, mevalonate 3-kinase; M3P, mevalonate 3-phosphate; PMVK, phosphomevalonate kinase; MDP, mevalonate 5-diphosphate; M5PDH, M5P dehydratase; AM5P, trans-anhydromevalonate 5-phosphate; AMPD, AM5P decarboxylase; MPD, M5P decarboxylase; IP, isopentenyl phosphate; M3PK, M3P 5-kinase; MBP, mevalonate 3,5-bisphosphate; MBD, MBP decarboxylase; DPMD, diphosphomevalonate decarboxylase.

Of the two known modern metabolic routes that generate the five carbon building-block precursor molecules from which all terpenoids are derived, the MVA pathway is generally used by eukaryotes and archaea (Zhao et al., 2013) and the MEP pathway is generally used by bacteria and plastids (Figures 1A,B; Rohmer, 2008; Zhao et al., 2013). The MEP pathway can theoretically generate more terpenoid product from the same starting material as the MVA pathway (Figures 1A,B; Rude and Schirmer, 2009; Whited et al., 2010). As such, the MEP pathway may have greater potential for producing terpenoids efficiently, but engineering efforts have yet to surmount several important barriers to increasing flux through the pathway.

The first enzyme of the MEP pathway, Dxs, is an initial bottleneck to carbon flux (Figure 1A; Harker and Bramley, 1999; Li and Wang, 2016). This bottleneck can be relieved by overexpression of Dxs, at which point IspG which converts methylerythritol 2, 4-cyclodiphosphate (MEcPP) to hydroxymethylbutenyl 4-diphosphate (HMBPP) becomes rate limiting (Zhou et al., 2012; Liu et al., 2014; Li et al., 2017; Volke et al., 2019; Khana et al., 2023). IspG and the next enzyme downstream, IspH, are both iron- sulfur cluster containing [4Fe-4S] proteins (Wang and Oldfield, 2014). Iron–sulfur cluster biogenesis and insertion into proteins is a complex process (Xu and Møller, 2011). After IspG and IspH have been loaded with iron–sulfur clusters, they require an external supply of reducing equivalents to function. Altogether, simple overexpression of IspG and IspH may not relieve the bottleneck because these proteins require extensive cellular processes in the generation and loading of iron–sulfur clusters and the maintenance of substrates for these proteins to function. Due to the barriers to increasing flux through IspG and IspH, identifying alternative enzymes to IspG and IspH would be advantageous for engineering of the MEP pathway.

Multiple routes that allow native or acquired circumvention of the first MEP pathway enzyme Dxs have been identified but to date no alternative routes circumventing IspG or IspH have been found (Figure 1A; Sauret-Güeto et al., 2006; Sangari et al., 2010; Erb et al., 2012; Perez-Gil et al., 2012; Warlick et al., 2012; Carretero-Paulet et al., 2013; Kirby et al., 2015). In contrast, multiple alternative routes for the MVA pathway for terpenoid precursor biosynthesis have been discovered, making it a pertinent demonstration of the potential for variations on a metabolic pathway to evolve using alternative enzymes and metabolites (Figure 1B; Hayakawa et al., 2018; Hoshino and Villanueva, 2023).

Here we define evolutionary flexibility as steps in metabolism for which multiple enzymatic solutions have evolved, as is seen in the MVA pathway (Figure 1B). In contrast, evolutionarily rigid metabolic steps are those for which alternative enzymes that would allow circumvention of that step have not evolved. To inform efforts to engineer the MEP pathway, we investigated the evolutionary diversity of terpenoid biosynthesis strategies in bacteria. We explored bacterial diversity by assessing an available database of bacterial genomes for terpenoid biosynthesis strategies and alternative enzymatic routes to steps in the MEP pathway. In particular, we asked if enzymes have evolved to circumvent IspG and IspH which pose known engineering constraints, or to Dxs, for which some alternatives exist. We identified bacteria with apparently incomplete MEP pathways as candidates for non-canonical terpenoid biosynthesis routes. Our study provides a comprehensive survey of the known bacterial species for non-canonical MEP pathways and underscores the evolutionary significance of IspG and IspH.

## 2. Materials and methods

### 2.1. Database of species and HMMER profiles

Fasta files of the NCBI database of reference and representative bacterial genomes available at the assembly level “complete chromosome” were downloaded (access date 6/19/2023). Profile Hidden Markov Models (pHMM) from the KOfam database (Aramaki et al., 2020) were downloaded from KEGG using genome.jp/ftp/db/kofam/ (access date 6/19/2023, KEGG release 106.0) as well as the “ko_list” file which contains adaptive score thresholds that define an ortholog match for each pHMM as determined by KOfam and described in more detail in section 2.3. The “ko_list” file was trimmed to only include the MEP and MVA genes of interest which increased computational analysis speed by limiting ortholog detection to only these genes. DxrII did not have a pHMM from KOfam, so we used hmmbuild to construct a pHMM model (Eddy, 2011) using the set of 7 genes predicted to be DxrII as annotated in NCBI’s conserved protein domain family in COG4091.

Full bioinformatic pipeline is available at https://github.com/bamarshall2/evolutionary_flexibility.

### 2.2. Open reading frame (ORF) and KofamScan for ortholog identification

For each genome, orfipy was used to identify ORFs of at least 150 nucleotides in length and generate an output of the peptide translation using the specifications “--min 150 --pep $file_name.fa” (Singh and Wurtele, 2021). All genomes were translated using standard codon table 1 except genera *Spiroplasma*, *Mycoplasma*, *Mycoplasmoides*, *Malacoplasma*, and *Ureaplasma* which were translated with codon table 4 according to their taxonomy using the additional orfipy argument “--table 4.” Kofam_scan tool was run on each orfipy output using the curated KO list of MEP and MVA genes of interest and pHMM profiles from the KOfam database. The kofam_scan command used was “./exec_annotation --format = detail-tsv -o ~ path_to_output/$output_file_name ~path_to_input/$input_file_name.”

### 2.3. Identification of orthologous genes

For Dxs, DxrI, IspE, IspDF, IspG, IspH, HMGS, HMGRI, HMGRII, MVK, DPMD, Rubisco-like protein MTRu-1P isomerase, and cupin type I MTXu-5P methylsulferase a gene ortholog was determined to be present if the similarity score from alignment exceeded the KOfam score threshold for identifying similarity (Aramaki et al., 2020). This task was completed using custom scripts, all code is available at GitHub.^1^

The computation of the KOfam score thresholds is described in detail in Aramaki et al. (2020). In brief, to compute the score threshold for a protein family in the KEGG Orthology database, the manually curated set of proteins is randomly divided into three groups, one for use as a positive training set and the other two for generation of the pHMM. Proteins that are not part of the protein family are used as a negative training dataset. Bit scores of alignments between the pHMM and the training data sets allows determination of a score threshold that maximizes the F measure which is the harmonic mean of precision and recall. The process of determining a threshold score is repeated three times by swapping the positive training set with one used to train the pHMM until each group of data has been used to both train and test the pHMMs. A final threshold score is then determined as the average of each iteration’s threshold score. For the proteins for which we used the score thresholds, the average F measure was 0.979 with MVK having the smallest F measure of 0.897 and DxrI having the highest F measure of 1. IspD and IspF occasionally exist as a bifunctional fusion protein called IspDF. We observed that presence/absence calls for these two proteins were sometimes incorrect when using the KOfam adaptive thresholds which is reflected by lower F measures for IspD (F measure = 0.720) and IspF (F measure = 0.704). These inaccurate calls may have been due to attempts to distinguish whether a monofunctional or bifunctional protein is present, which requires high thresholds for alignment scores and causes reduced accuracy. For our purposes, identification of an IspD or IspF domain is of key relevance, but whether it is in a monofunctional or bifunctional version of the protein is not. To best identify IspD and IspF, we use the KOfam adaptive threshold for identification of bifunctional IspDF (F measure = 0.997), and for IspD or IspF we set a cutoff threshold of ORFs with alignment e-values of less than 1^−10^. The KOfam database did not have an adaptive threshold cutoff for DxrII for determination of orthology. For DxrII, we set the score cutoff at alignments with e-values of less than 1^−10^ as well.

To determine a species’ MEP and MVA gene status, we sorted for presence and absence using Excel. Full table output of gene presence or absence is available in Supplementary Table S1.

### 2.4. Determining MVA pathway status

Diversity exists in the lower part of the MVA pathway (Figure 1B) but complete characterization of the MVA pathway was not the goal of this research so determination of MVA pathway presence was more relaxed than analyses for the MEP pathway. For the MVA pathway, if 2 out of 4 genes (encoding HMGS, HMGRI/II, MVK, and DPMD) were present, the organism was considered to possess the MVA pathway.

### 2.5. Phylogenetic tree building

Species trees were constructed using Genome Taxonomy Database (GTDB) master tree (Parks et al., 2018) and only the species included in this study were retained. The GTDB master tree included 2,210 of the 4,400 species (~50%) in the presented analyses, so the phylogenetic trees show half of the species analyzed.

For protein trees, the protein sequences corresponding to the entries from the species tree were extracted and aligned by MAFFT (Katoh et al., 2002) using default parameters with iteration refinement over 1,000 cycles. Columns with >95% gaps were removed using TrimAL (Capella-Gutiérrez et al., 2009). These sequences were used to build the phylogenetic tree for the MEP pathway proteins. A maximum-likelihood phylogenetic tree was constructed for each of the protein using IQ-Tree 2 (Nguyen et al., 2015; Minh et al., 2020) and ModelFinder plus (Kalyaanamoorthy et al., 2017) was implemented for selecting the best-fit evolutionary model. Branch support values were calculated using the Shimodaira–Hasegawa approximate likelihood-ratio test (SH-aLRT) with 1,000 bootstrap replicates and 1,000 ultrafast bootstrap (UFBoot) replicates optimized by the nearest neighbor interchange (NNI). The phylogenetic trees were visualized using FigTree v1.4.4 (Rambaut, 2018). The reconstructed trees were rooted using midpoint rooting and annotated in R studio (Farris, 1972).

## 3. Results

### 3.1. Some bacteria use a non-canonical MEP pathway lacking Dxs

**Figure 2:**
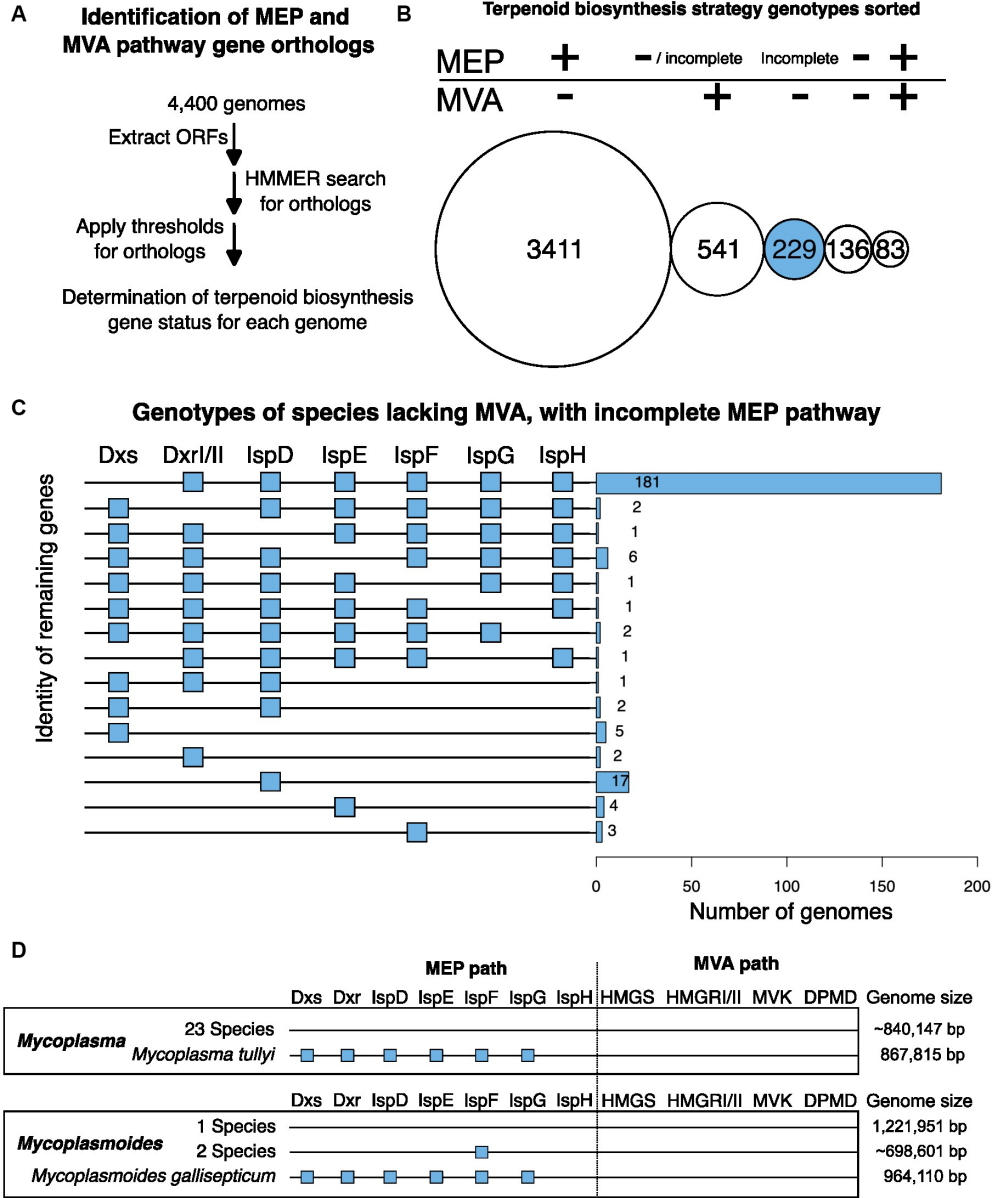
*In silico* identification of terpenoid biosynthesis metabolic pathways. **(A)** Bioinformatic analysis pipeline. The HMMER search for orthologs used profile Hidden Markov Models (pHMM) from the KOfam database (Aramaki et al., 2020). For ortholog identification cutoff values of genes Dxs, DxrI, IspE, IspDF, IspG, and IspH, we relied on the KOfam database of adaptive score thresholds determined for each gene (Aramaki et al., 2020). For DxrII, IspD, and IspF we defined orthologs as alignments to the pHMM with e-values lower than 1^−10^. See Methods for more details. **(B)** Terpenoid biosynthesis strategy genotypes sorted. Circle areas are scaled to the total number of species for each genotype. “+” indicates all enzymes of the MEP pathway or at least two enzymes of the MVA pathway were detected. “– “indicates none of the enzymes of the MEP pathway or fewer than two enzymes of the MVA pathway were detected. Incomplete indicates that only some of the enzymes of the MEP pathway were detected. For the 541 MVA+ species, some have no detectable MEP orthologs while others do. This is indicated by the “−/incomplete” description of the MEP pathway. **(C)** Bar chart of 229 candidate species for alternative MEP pathways with visual descriptor of genotype on the left. A blue box indicates the presence of the gene whereas no box indicates a lack of that gene. **(D)** Visual representation of MEP and MVA genes present in *Mycoplasma* and *Mycoplasmoides*. The presence of a blue square indicates the identification of a gene ortholog. The lack of a square indicates that no ortholog was identified. Genome sizes are displayed to the right, with average genome sizes indicated with “~”.

To find candidate species for a non-canonical MEP pathway, we started with an available database of representative bacterial genomes (O’Leary et al., 2016) and extracted all open reading frames (ORFs) from these genomes (Singh and Wurtele, 2021). Next, we used a HMMER based search tool called KofamScan for identification of orthologs to MEP and MVA genes (Figure 2A; Potter et al., 2018; Aramaki et al., 2020).

Of 4,400 species in the database, 3,411 (78%) had all 7 MEP genes detected (Dxs, DxrI/II, IspD, IspE, IspF, IspG, and IspH) but lack the MVA pathway according to our criteria (see Methods) (Figure 2B). Some species (541; 12%) had the MVA pathway and no MEP pathway genes or an incomplete MEP pathway detected whereas others (83; 1.8%) had all genes for the MEP pathway and at least two MVA pathway genes. Of 136 species (3.1%) that lacked a complete set of identifiable orthologs for both the MEP and MVA pathways, many had small genomes with a median size of 843,495 base pairs and included known endosymbiotic species *Mycoplasma*, *Rickettsia*, and *Spiroplasma* (Eberl et al., 2004). The remaining 229 species (5.2%) had at most one MVA gene and an incomplete MEP pathway where at least one gene of Dxs, DxrI/II, IspD, IspE, IspF, IspG, or IspH appeared to be missing making them candidates for species using a non-canonical MEP pathway (Figure 2B).

Of these 229 candidates, most were missing Dxs only (181, 79%) (Figure 2C) and thus might use an alternative to Dxs. One such potential alternative is the 5-methylthioadenosine (MTA) isoprenoid shunt first identified in *Rhodospirillum rubrum* (Figure 1; Erb et al., 2012). The MTA-isoprenoid shunt uses the enzymes Rubisco-Like protein MTRu-1P isomerase and Cupin type I protein MTXu-5P methylsulferase to convert polyamine biosynthesis intermediate MTA to isoprenoid biosynthesis intermediate 1-deoxy-D-xylulose 5-phosphate (DXP) (Warlick et al., 2012). For the candidate non-canonical MEP genomes lacking Dxs, 4 (2%) had only the MTXu-5P methylsulfurase and 5 (3%) had both the MTXu-5P methylsulfurase and the MTRu-1P isomerase (Supplementary Table S2). In these species, it is possible the MTA-isoprenoid shunt may substitute for Dxs, but this hypothesis will require further testing.

For the remaining 172 candidate species (95%) missing Dxs, the specific mechanism that allows the absence of Dxs requires further investigation to determine. Regardless of the mechanism, the identification of candidate species that contain all other MEP enzymes except Dxs suggest that the MEP pathway has evolutionary flexibility for the enzymatic activity that Dxs provides.

### 3.2. IspG and IspH are evolutionarily rigid

Dxs is the only MEP pathway enzyme for which we identified a subset of candidate species that lack Dxs suggesting that an alternative strategy that circumvents Dxs has evolved. For IspG and IspH, there were no promising candidates for identification of alternative MEP pathways that circumvent one or both enzymes (Figure 2C). Thus, we define IspG and IspH as evolutionary rigid.

The single candidate genome lacking IspG only was *Lelliotia steviae* (Figure 2C). When investigated, we found that the fasta file for this species was 1 million base pairs shorter than the sequence originally published and that other closely related *Lelliottia* species all had IspG present (Supplementary Table S4). Thus, we suggest the full *Lelliotia steviae* genome likely encodes IspG but that it may be in the 1 million missing base pairs. We interpret this single genome lacking IspG to be indicative of the level of inaccuracy within a large, collaborative database of species.

The two species lacking IspH only were *Mycoplasma tullyi* and *Mycoplasmoides gallisepticum* (Figures 2C,D). Both species have genomes smaller than 1 million base pairs and all other *Mycoplasma* and *Mycoplasmodies* included in this database had neither the MVA nor the MEP pathway consistent with all being endosymbionts and lacking terpenoid biosynthesis metabolisms (Figure 2D; Eberl et al., 2004). It appears that though closely related *Mycoplasma* and *Mycoplasmoides* species have evolved to rely on host isoprenoid biosynthesis, *Mycoplasma tullyi* and *Mycoplasmoides gallisepticum* have retained a near complete MEP pathway despite having comparably small genomes. *Mycoplasmoides gallisepticum* has been demonstrated to generate MEP pathway intermediate HMBPP (Eberl et al., 2004) though the essentiality of the MEP pathway in either organism remains to be confirmed. With only two species that are outliers in their genera, they do not constitute a compelling lead for identifying an alternative enzyme to IspH. It is possible that these species are making use of a near complete MEP pathway that ends with the generation of HMBPP and it is also possible that they do possess an alternative enzyme that circumvents IspH. These two species may have their own interesting evolutionary rationale for maintaining a nearly complete MEP pathway, but it will require further study to understand. The remaining 45 candidate species have no compelling patterns that suggest the presence of a non-canonical MEP pathway that would aid in the engineering of the MEP pathway (Figure 2B).

We conclude that an alternative enzyme to Dxs probably exists and is in use in some of the 181 candidate species lacking Dxs. We interpret this to mean that an alternative to Dxs has probably evolved making it evolutionarily flexible. Meanwhile, given our approach, we cannot identify any species that may be using alternative enzymes to IspG or IspH.

### 3.3. Phylogenetic distribution of species lacking Dxs

**Figure 3:**
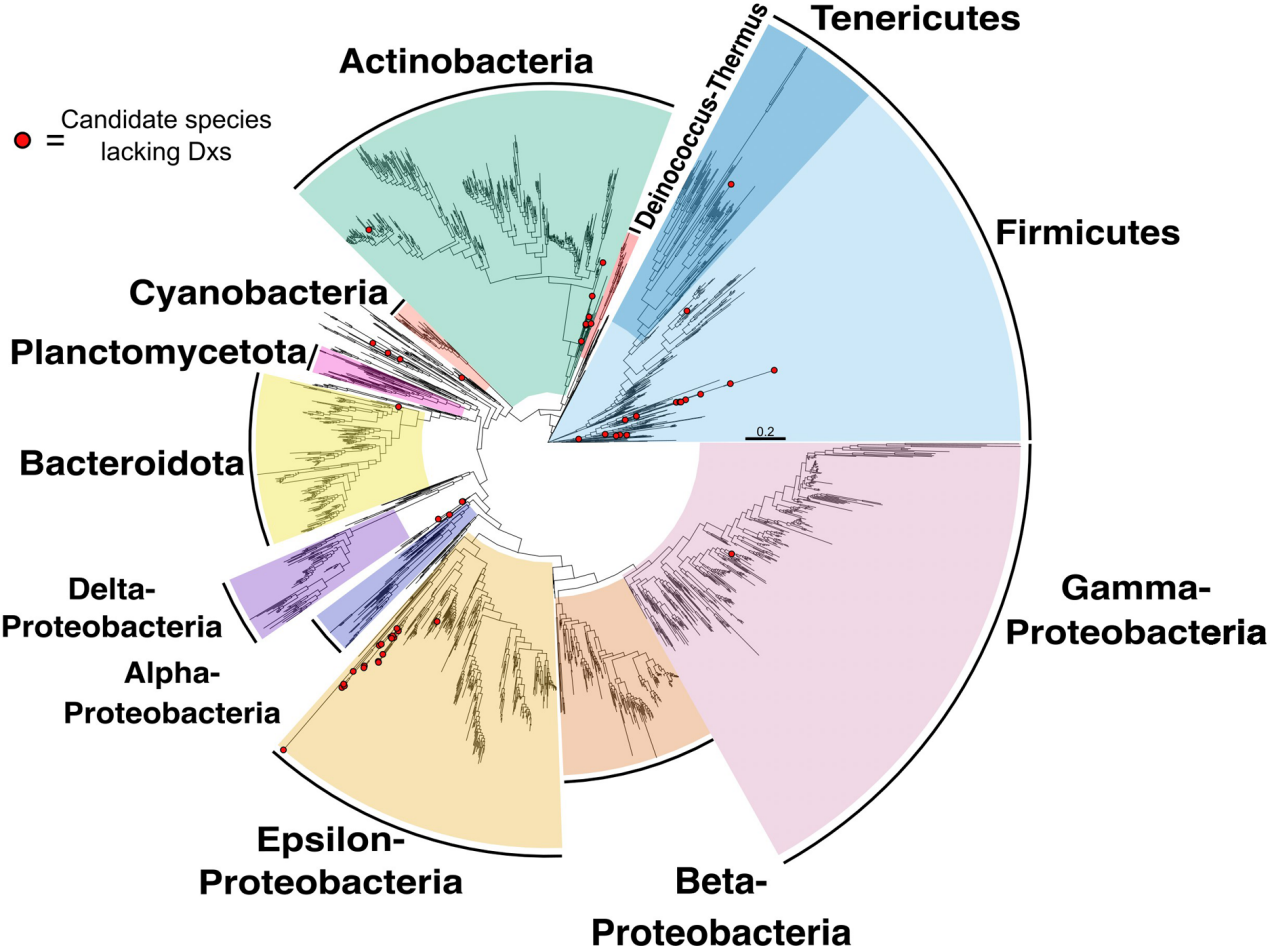
Species tree highlighting candidate species lacking Dxs. Species tree of species included in the Genome Taxonomy Database (GTDB) and in the NCBI reference and representative database of complete genomes analyzed in this report (see Methods for details). Species with red circles at tip lack Dxs.

Candidate species that might encode a non-canonical MEP pathway that lack Dxs are distributed across different phylogenetic groups (Figure 3). Species lacking Dxs can be found in the phyla Firmicutes, Actinobacteria, Alpha-proteobacteria and others. A complete list of these species is presented in Supplementary Figure S3. These species may use the same or different strategies to circumvent Dxs, but additional analysis will be required to identify these pathways.

### 3.4. Within a genus terpenoid biosynthesis strategies vary at the species level

**Figure 4:**
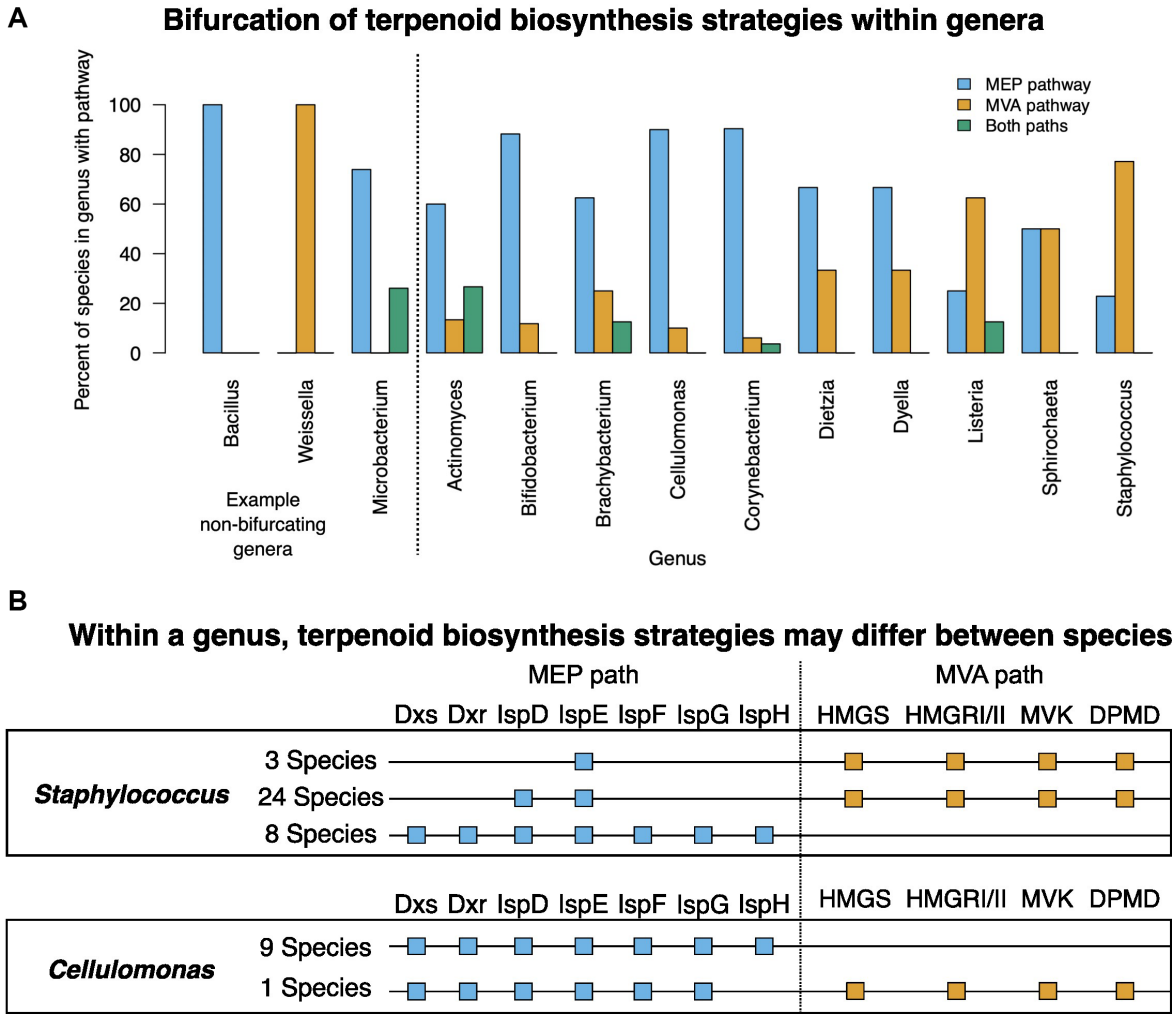
Genera with species possessing the MEP and MVA pathways. **(A)** All genera with bifurcated terpenoid biosynthesis strategies. Representative non-bifurcating genera *Bacillus*, *Weissella*, and *Microbacterium* are shown for comparison on the left. Some of the species summarized in this bar chart have enzymes from the alternative terpenoid biosynthesis pathway. For complete description of each species’ terpenoid biosynthesis gene status, see Supplementary Table S1. **(B)** MEP and MVA genotypes of *Staphylococcus* and *Cellulomonas* species as examples showing that within a single genus, species may bifurcate with regards to their terpenoid biosynthesis strategy.

Generally, all species in a genus use either the MEP or MVA pathway (e.g., *Bacillus* and *Weissella*; Figure 4A). Another common pattern is for species to possess one complete pathway, and some of the enzymes of the other pathway. In other genera, most species use the same isoprenoid biosynthesis pathway although a subset encode enzymes for both pathways (e.g., *Microbacterium*; Figure 4A). For 10 genera with multiple represented species, terpenoid biosynthesis strategies are bifurcated (Figure 4A). In these genera, some species use one terpenoid biosynthesis strategy whereas other species use the other terpenoid biosynthesis strategy.

For example, *Staphylococcus* was represented by 35 species in the database, 27 of which (77%) used the MVA pathway and possess one or two MEP pathway enzymes whereas the remaining 8 species (23%) encode complete MEP pathways but no MVA pathway enzymes (Figure 4B) (Misic et al., 2016). In *Cellulomonas*, 9 of the species encode a complete MEP pathway with no MVA enzymes but a single species, *Cellulomonas taurus,* encodes an incomplete MEP pathway and a complete MVA pathway (Figure 4B). The MVA pathway genes in *Cellulomonas taurus* are in two operons in the genome, roughly 1 million base pairs apart.

These data suggest that selective pressure exists to retain a terpenoid biosynthesis strategy, but that a species may use MEP, MVA, or both based on evolutionary trajectory.

### 3.5. IspG protein tree differs from species tree and the three-domain IspG proteins cluster together

**Figure 5:**
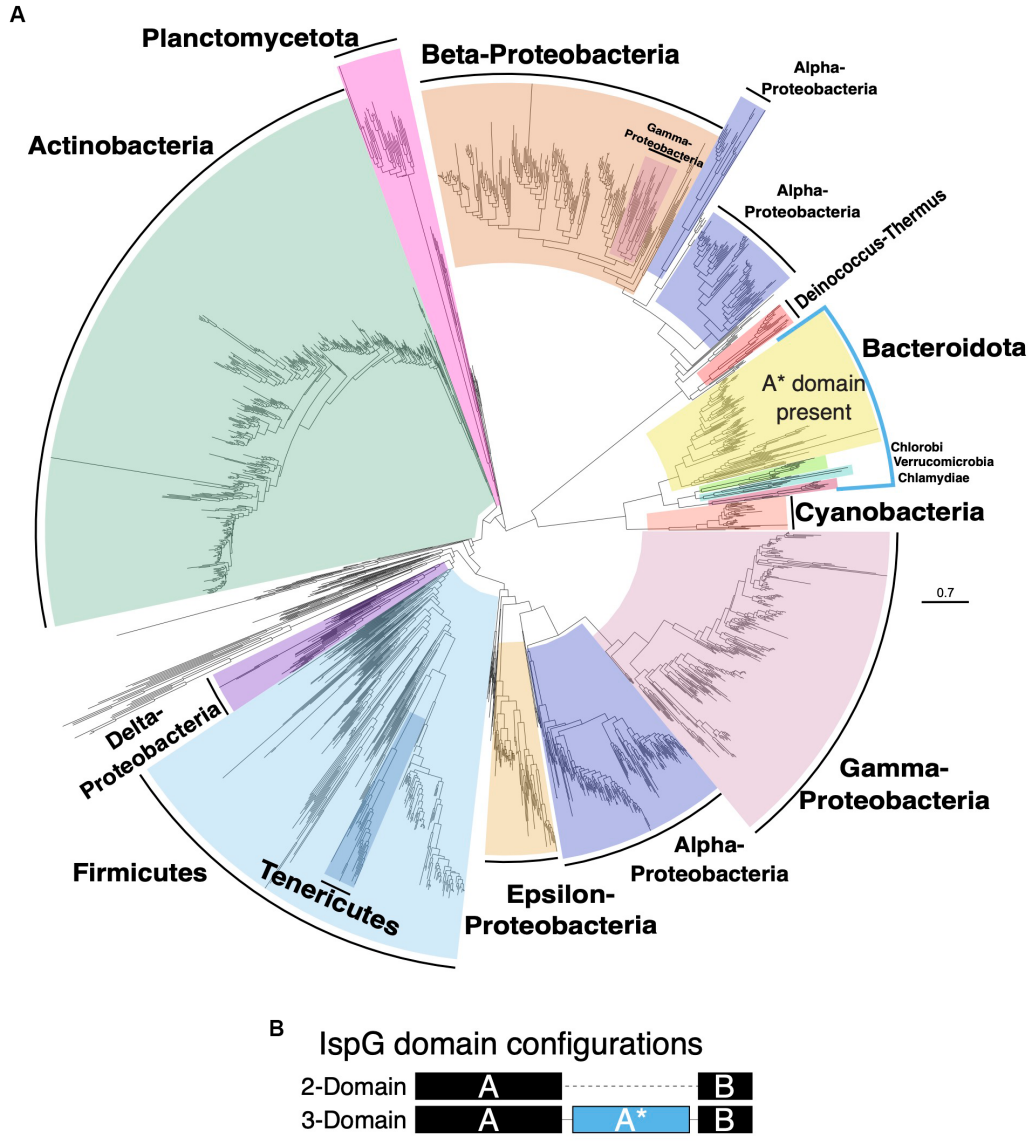
Protein tree of IspG. **(A)** Phylogenetic tree based on alignment of IspG protein identified in each organism. Species that have A* domain present in their IspG are highlighted in blue. **(B)** IspG domain configurations. IspG may either have two domains A and B or three domains A, A* and B.

To understand the impact of horizontal gene transfer on the MEP pathway in bacteria, we generated evolutionary trees from protein sequences for Dxs, DxrI, IspE, IspG, and IspH (Supplementary Figure S5). The Dxs, DxrI, IspE, IspG, and IspH trees differ from the species tree in that Actinobacteria have clustered with Proteobacteria in the protein trees instead of Bacteroidota as was the case of the species tree (Figures 3, 5A and Supplementary Figure S5 ). Also, a notable feature for these protein trees is the paraphyletic nature of the Proteobacteria phylum, which is most clearly observed in the IspG tree. In the IspG protein tree, the Proteobacterial phylum is split into two groups; one Proteobacterial group clusters with Bacteroidota and the other with Firmicutes. Further, the Proteobacterial phylum is more widely distributed in the IspG protein tree with some γ-Proteobacteria clustering within β-Proteobacteria and the α-Proteobacteria being split.

IspG is generally a two-domain protein in Bacteria, but a third domain called A* may be present (Figure 5B; Liu et al., 2012). The A* domain has a TIM barrel fold, but its exact role has yet to be elucidated (Liu et al., 2012). The phyla Bacteroidota, Chlorobi, Verrucomicrobia, and Chlamydiae all possess the A* domain and cluster together (Figures 5A,B). Plancomycetota, Verrucomicrobia, and Chlamydiae are the major phyla that constitute superphylum PVC (Wagner and Horn, 2006), however only Verrucomicrobia and Chlamydiae possess the three-domain form of IspG whereas Planctomycetota possess the 2-domain form. This splitting of the PVC superphylum with regards to IspG has been illustrated previously (Zeng and Dehesh, 2021). Absence of the three-domain form of IspG only in Planctomycetota of the PVC superphylum could be due to horizontal gene transfer as the phylogenetic placement of the Plancomycetota IspG sequence in the protein tree is not congruent with the PVC clade in the species tree. An alternative possibility is that the A* domain evolved in a common ancestor of Bacteroidota, Plancomycetota, Verrucomicrobia, and Chlamydiae that was then lost in Planctomycetota. With the data presented here, these two possibilities cannot be distinguished.

Based on the differences between the MEP protein trees and the species tree, MEP pathway inheritance is not strictly vertical. Therefore, we suggest that horizontal gene transfer may have played a role in the evolution of this metabolic pathway.

## 4. Discussion

In this study, we assessed the MEP and MVA pathways and protein diversity across bacteria. Our goal was to inform options for engineering the precursor pathways for microbial terpenoid biomanufacturing by determining if alternatives exist for some steps in precursor pathways. In particular, we were interested in alternatives to Dxs and whether non-canonical MEP pathways can circumvent the IspG and IspH enzymes, which are known to pose major engineering challenges. To achieve this goal, we identified genomes lacking the MVA pathway, with an incomplete MEP pathway referred to as candidate species for a non-canonical MEP pathway. We found that Dxs is evolutionarily flexible and that alternatives may exist whereas for IspG and IspH we could not identify any candidate species that appear to circumvent these enzymes. These results may aid the ongoing quest to understand early evolution of terpenoid biosynthetic pathways.

Identified alternatives to the canonical MEP enzymes include DxrII, the MTA-isoprenoid shunt, and mutations that arise when Dxs is knocked out (Figure 1A; Sauret-Güeto et al., 2006; Sangari et al., 2010; Erb et al., 2012; Perez-Gil et al., 2012; Warlick et al., 2012; Carretero-Paulet et al., 2013; Kirby et al., 2015). The suppressor mutations that have been documented to arise when Dxs is knocked out include mutations in AceE, RibB, or overexpression of YajO. Altogether, there are multiple routes that circumvent Dxs but alternatives pathways to circumvent IspG or IspH have not yet been identified.

We identified 181 candidate species for a non-canonical MEP pathway missing Dxs. In contrast, we identified fewer than 40 species missing IspG, IspH, or both in combination with other MEP enzymes and no compelling patterns among these species (Figure 2C). We conclude that within this database of 4,400 species, all MEP utilizing species possess IspG and IspH as part of their MEP pathways. On the other hand, some of the 181 candidate species missing Dxs likely have an alternative MEP pathway strategy. This result would align with previous research where alternative routes for circumventing Dxs have been identified (see above).

Taken together, our results suggest that within the MEP pathway, evolutionary flexibility exists for Dxs. Alternative strategies to circumvent Dxs have likely evolved whereas IspG and IspH are evolutionarily rigid and no alternatives appear to have evolved or can be identified at this time. DxrI/II, IspD, IspE and IspF are also evolutionarily rigid as they appear to be retained by essentially all species using the MEP pathway. Whether the evolutionary rigidity of IspG, IspH, DxrI/II, IspD, IspE and IspF is due to a functional constraint hindering biochemical ability or due to the lack of sequence diversity available for the enzyme to explore under an evolving landscape remains an open question.

The evolutionary rigidity of enzymes in the MEP pathway may be valuable for unraveling the evolution of terpenoid biosynthesis. Analysis of the protein phylogenetic trees for the MEP enzymes was consistent with the possibility that horizontal gene transfer has played a role in the evolution of these enzymes (Figure 5; Supplementary Figure S5). The role of horizontal gene transfer in extant species is highlighted in Figure 4 where within some genera bifurcation of the terpenoid biosynthesis strategies has occurred.

Species retaining all MEP enzymes except Dxs, are distributed across different phylogenetic groups (Figure 3). It is possible that these species acquired the same alternative to Dxs via horizontal gene transfer. Another possibility is that multiple strategies have evolved. Further work will be required to determine how these species circumvent Dxs as well as the relationship of these metabolic routes to each other. Possible explanations for the lack of Dxs include incomplete sequencing, promiscuous activity of related enzymes at levels adequate to support loss of Dxs, or an alternative enzyme evolved that is functionally equivalent to Dxs which should be sought.

A limitation of our bioinformatic approach is that it will only identify alternative MEP pathways in species lacking redundancy. Species with a complete MEP or MVA pathway in addition to an alternative non-canonical MEP pathway would not be identified. Additionally, we only surveyed the current database of bacterial species, which is inherently biased toward culturable bacteria and does not capture the full diversity of life (Hugenholtz and Pace, 1996; Hug et al., 2016). It is possible that in organisms not yet sequenced, alternative MEP pathways may exist. Finally, we note that sequence-based identification of orthologs could miss functional enzymes that lack sequence similarity. Regardless, our results provide valuable insights into MEP pathway constraints and identify a set of species that are promising candidates for an alternative MEP pathway that circumvents Dxs.

Our findings also inform metabolic engineering of the MEP pathway in two ways. First regarding Dxs, we find that alternative enzymes to Dxs may exist. As Dxs governs MEP pathway flux via feedback regulation (Banerjee et al., 2013) and requires pyruvate and glyceraldehyde-3-phosphate as the MEP pathway substrates, it would be of value to metabolic engineers to identify Dxs alternatives. An alternative enzyme to Dxs may consume different substrates or avoid regulation. For IspG and IspH, ongoing efforts to engineer these enzymes to increase their flux are justified, as no alternatives to their role in the MEP pathway were apparent.

The development of sequencing technologies has enabled the determination of unprecedented numbers of bacterial genomes. This database of bacterial genomes is a repository of knowledge with astounding potential. Here, we leveraged the current repository of bacterial genomes as a resource for identifying alternative enzymes to enable engineering of a key metabolic pathway. Future efforts in microbial engineering should also utilize the database of bacterial genomes to search for naturally evolved alternatives for cellular metabolism that go beyond currently characterized literature.

## Data availability statement

The original contributions presented in the study are included in the article/Supplementary material, further inquiries can be directed to the corresponding authors.

## Author contributions

BM: Conceptualization, Data curation, Investigation, Methodology, Software, Visualization, Writing – original draft, Writing – review & editing. KA: Software, Visualization, Writing – review & editing. MW: Software, Supervision, Writing – review & editing. BK: Conceptualization, Funding acquisition, Supervision, Writing – review & editing. RL: Conceptualization, Funding acquisition, Supervision, Writing – review & editing.

## Footnotes

^1^ https://github.com/bamarshall2/evolutionary_flexibility
