# Supplementary material for: Evolutionary flexibility and rigidity in the bacterial methylerythritol phosphate (MEP) pathway
Source: Front Microbiol. 2023 Nov 8;14:1286626. doi: 10.3389/fmicb.2023.1286626 (PMC10663253; doi:10.3389/fmicb.2023.1286626)
Supplement: Supplementary file 1 [file Data_Sheet_1.zip › Supplementary File S1.DOCX]

List of candidate species lacking Dxs

Acetobacterium woodii DSM 1030

Acidilutibacter cellobiosedens

Alkalibacter rhizosphaerae

Anaerococcus mediterraneensis

Anaerococcus obesiensis

Anaerococcus vaginalis

Anaplasma centrale str. Israel Anaplasma marginale subsp. centrale

Anaplasma marginale str. Florida

Anaplasma ovis str. Haibei

Anaplasma phagocytophilum str. JM

Atribacter laminatus

Candidatus Bipolaricaulis anaerobius

Candidatus Fokinia solitaria

Candidatus Koribacter versatilis Ellin345

Candidatus Neoehrlichia mikurensis

Chloracidobacterium sp. MS 40 45

Chloracidobacterium thermophilum B

Chloracidobacterium validum

Chordicoccus furentiruminis

Christensenella minuta

Dialister hominis

Dialister massiliensis

Egibacter rhizosphaerae

Ehrlichia canis str. Jake

Ehrlichia chaffeensis str. West Paces

Ehrlichia muris AS145

Ehrlichia ruminantium heartwater rickettsia

Eubacterium callanderi

Eubacterium limosum

Eubacterium maltosivorans

Euzebya pacifica

Ezakiella massiliensis

Fenollaria massiliensis

Gudongella oleilytica

Keratinibaculum paraultunense

Lactococcus carnosus

Lactococcus paracarnosus

Lactococcus raffinolactis

Luteitalea pratensis

Megasphaera elsdenii

Megasphaera hexanoica

Megasphaera stantonii

Miniphocaeibacter halophilus

Murdochiella vaginalis

Mycolicibacterium pulveris Mycobacterium pulveris

Ndongobacter massiliensis

Neorickettsia findlayensis

Neorickettsia helminthoeca str. Oregon

Neorickettsia risticii str. Illinois

Neorickettsia sennetsu str. Miyayama

Olsenella timonensis

Paludibaculum fermentans

Paraburkholderia dokdonensis

Paracoccus mutanolyticus

Peptoniphilus harei

Peptoniphilus ivorii

Rhodothermus marinus DSM 4252

Rubrobacter indicoceani

Rubrobacter marinus

Rubrobacter tropicus

Rubrobacter xylanophilus DSM 9941

Salinibacter ruber

Salinicola tamaricis

Sphaerochaeta associata

Sphaerochaeta coccoides DSM 17374

Sphaerochaeta globosa str. Buddy

Sphaerochaeta pleomorpha str. Grapes

Spiroplasma clarkii

Tepidanaerobacter acetatoxydans Re1

Thermodesulfovibrio yellowstonii DSM 11347

Thermosediminibacter oceani DSM 16646

Veillonella nakazawae

Veillonella parvula

Wolbachia endosymbiont group A of Acrocera orbiculus

Wolbachia endosymbiont group A of Ancistrocerus nigricornis

Wolbachia endosymbiont group A of Andrena dorsata

Wolbachia endosymbiont group A of Andrena haemorrhoa

Wolbachia endosymbiont group A of Anomoia purmunda

Wolbachia endosymbiont group A of Anoplius nigerrimus

Wolbachia endosymbiont group A of Apoderus coryli

Wolbachia endosymbiont group A of Bibio marci

Wolbachia endosymbiont group A of Bombylius major

Wolbachia endosymbiont group A of Calamotropha paludella

Wolbachia endosymbiont group A of Cheilosia soror

Wolbachia endosymbiont group A of Coremacera marginata

Wolbachia endosymbiont group A of Ectemnius continuus

Wolbachia endosymbiont group A of Endotricha flammealis

Wolbachia endosymbiont group A of Epagoge grotiana

Wolbachia endosymbiont group A of Epirrhoe alternata

Wolbachia endosymbiont group A of Epistrophe grossularia

Wolbachia endosymbiont group A of Eupithecia tripunctaria

Wolbachia endosymbiont group A of Gymnosoma rotundatum

Wolbachia endosymbiont group A of Hylaeus communis

Wolbachia endosymbiont group A of Icerya purchasi

Wolbachia endosymbiont group A of Lasioglossum lativentre

Wolbachia endosymbiont group A of Lasioglossum morio

Wolbachia endosymbiont group A of Macropis europaea

Wolbachia endosymbiont group A of Merzomyia westermanni

Wolbachia endosymbiont group A of Nomada fabriciana

Wolbachia endosymbiont group A of Phalera bucephala

Wolbachia endosymbiont group A of Pheosia gnoma

Wolbachia endosymbiont group A of Philonthus cognatus

Wolbachia endosymbiont group A of Protocalliphora azurea

Wolbachia endosymbiont group A of Rhinocyllus conicus

Wolbachia endosymbiont group A of Scambus nigricans

Wolbachia endosymbiont group A of Sicus ferrugineus

Wolbachia endosymbiont group A of Sphaerophoria taeniata

Wolbachia endosymbiont group A of Sphecodes monilicornis

Wolbachia endosymbiont group A of Sympetrum striolatum

Wolbachia endosymbiont group A of Tiphia femorata

Wolbachia endosymbiont group A of Trypoxylon clavicerum

Wolbachia endosymbiont group A of Yponomeuta plumbellus

Wolbachia endosymbiont group B of Agriphila straminella

Wolbachia endosymbiont group B of Apotomis betuletana

Wolbachia endosymbiont group B of Apotomis turbidana

Wolbachia endosymbiont group B of Archips podanus

Wolbachia endosymbiont group B of Athalia cordata

Wolbachia endosymbiont group B of Campaea margaritaria

Wolbachia endosymbiont group B of Camptogramma bilineatum

Wolbachia endosymbiont group B of Catoptria pinella

Wolbachia endosymbiont group B of Celastrina argiolus

Wolbachia endosymbiont group B of Chorthippus parallelus

Wolbachia endosymbiont group B of Colias croceus

Wolbachia endosymbiont group B of Emmelina monodactyla

Wolbachia endosymbiont group B of Endotricha flammealis

Wolbachia endosymbiont group B of Episyrphus balteatus

Wolbachia endosymbiont group B of Erynnis tages

Wolbachia endosymbiont group B of Eucosma cana

Wolbachia endosymbiont group B of Eupeodes latifasciatus

Wolbachia endosymbiont group B of Euphydryas aurinia

Wolbachia endosymbiont group B of Hylaea fasciaria

Wolbachia endosymbiont group B of Ischnura elegans

Wolbachia endosymbiont group B of Lycaena phlaeas

Wolbachia endosymbiont group B of Melanostoma mellinum

Wolbachia endosymbiont group B of Nymphalis c-album

Wolbachia endosymbiont group B of Pammene fasciana

Wolbachia endosymbiont group B of Pandemis corylana

Wolbachia endosymbiont group B of Parapoynx stratiotata

Wolbachia endosymbiont group B of Pararge aegeria

Wolbachia endosymbiont group B of Phalera bucephala

Wolbachia endosymbiont group B of Pheosia tremula

Wolbachia endosymbiont group B of Polyommatus icarus

Wolbachia endosymbiont group B of Protocalliphora azurea

Wolbachia endosymbiont group B of Pyrgus malvae

Wolbachia endosymbiont group B of Rhopobota naevana

Wolbachia endosymbiont group B of Thymelicus sylvestris

Wolbachia endosymbiont group B of Watsonalla binaria

Wolbachia endosymbiont group B of Xestia c-nigrum

Wolbachia endosymbiont of Aedes aegypti

Wolbachia endosymbiont of Aedes albopictus

Wolbachia endosymbiont of Anopheles demeilloni

Wolbachia endosymbiont of Brugia pahangi

Wolbachia endosymbiont of Carposina sasakii

Wolbachia endosymbiont of Chrysomya megacephala

Wolbachia endosymbiont of Cimex lectularius

Wolbachia endosymbiont of Corcyra cephalonica

Wolbachia endosymbiont of Cruorifilaria tuberocauda

Wolbachia endosymbiont of Ctenocephalides felis wCfeJ

Wolbachia endosymbiont of Ctenocephalides felis wCfeT

Wolbachia endosymbiont of Delia radicum

Wolbachia endosymbiont of Dipetalonema caudispina

Wolbachia endosymbiont of Dirofilaria Dirofilaria immitis

Wolbachia endosymbiont of Drosophila ananassae

Wolbachia endosymbiont of Drosophila innubila

Wolbachia endosymbiont of Drosophila mauritiana

Wolbachia endosymbiont of Drosophila melanogaster

Wolbachia endosymbiont of Drosophila pseudotakahashii

Wolbachia endosymbiont of Drosophila santomea

Wolbachia endosymbiont of Drosophila simulans wHa

Wolbachia endosymbiont of Drosophila yakuba

Wolbachia endosymbiont of Folsomia candida

Wolbachia endosymbiont of Litomosoides sigmodontis

Wolbachia endosymbiont of Oedothorax gibbosus

Wolbachia endosymbiont of Oryzaephilus surinamensis

Wolbachia endosymbiont of Ostrinia furnacalis

Wolbachia endosymbiont of Ostrinia scapulalis

Wolbachia endosymbiont of Phyllotreta cruciferae

Wolbachia endosymbiont of Psylliodes chrysocephala

Wolbachia endosymbiont of Spodoptera picta

Wolbachia endosymbiont of Wiebesia pumilae

Wolbachia endosymbiont strain TRS of Brugia malayi
