# Supplementary material for: Evolutionary flexibility and rigidity in the bacterial methylerythritol phosphate (MEP) pathway
Source: Front Microbiol. 2023 Nov 8;14:1286626. doi: 10.3389/fmicb.2023.1286626 (PMC10663253; doi:10.3389/fmicb.2023.1286626)

Supplementary Material

# Supplementary Data

## Supplementary table S1 – Terpenoid biosynthesis gene status of all species

Excel document of gene presence or absence for all species analyzed in this report. Identification of an ortholog in a species is denoted by a ‘2’ while the lack of identification of an ortholog is denoted with a ‘1’. Criteria for ortholog identification is described in the **Methods**. In brief, Dxs, DxrI, IspDF, IspE, IspG, and IspH orthologs are identified if an ORF similarity score to the KOfam pHMM exceeds the adaptive threshold (Aramaki et al., 2020). For DxrII, IspD, and IspF the cutoff threshold were ORF’s with e-values that were smaller than 1^-10^.

## Supplementary table S2 – Candidate species lacking Dxs with potential MTA-isoprenoid shunt

Candidate genomes for alternative MEP pathway lacking Dxs and having at least one gene involved in MTA-isoprenoid shunt. These are the 9 species that have fewer than 2 enzymes from the MVA pathway which are missing Dxs and also have at least part of the MTA-isoprenoid shunt.

## Supplementary file S1 – All candidate species lacking Dxs

List of candidate species lacking Dxs. This the complete list of 181 species that have fewer than 2 MVA pathway enzymes which are also missing Dxs while encoding the rest of the MEP pathway enzymes.

## Supplementary table S3 – *Lelliotia* species’ IspG information

Table of *Lelliotia* species’ information. Similarity to *Lelliotia steviae* based on Lin et al., 2022. We could not identify IspG in *Lelliotia steviae* but display in this table that closely related species do have IspG and a large difference in genome size to the available genome file for *L. steviae*.

## Supplementary figure S1 – Protein trees for all MEP proteins

Species tree assembled from genome taxonomy database (GTDB) and Protein trees assembled from protein alignments for MEP proteins Dxs, DxrI, IspE, IspG, and IspH.


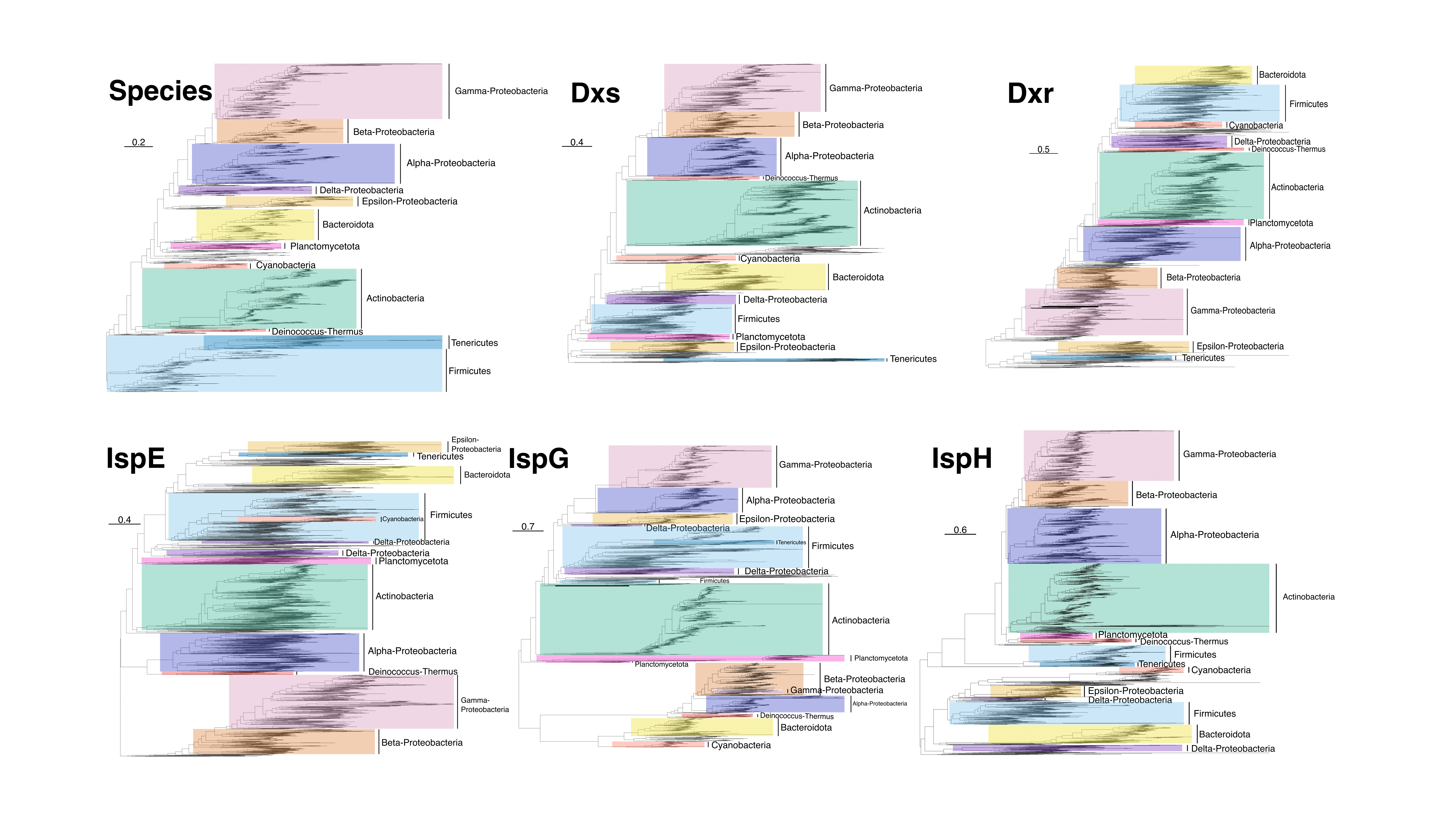

Supplement: Supplementary file 1 [file Data_Sheet_1.zip › Supplementary material captions.DOCX]
